# Mercapto-pyrimidines are reversible covalent inhibitors of the papain-like protease (PLpro) and inhibit SARS-CoV-2 (SCoV-2) replication†

**DOI:** 10.1039/d3ra01915b

**Published:** 2023-06-12

**Authors:** Teena Bajaj, Eddie Wehri, Rahul K. Suryawanshi, Elizabeth King, Kundan Singh Pardeshi, Kamyar Behrouzi, Zahra Khodabakhshi, Ursula Schulze-Gahmen, G. Renuka Kumar, Mohammad R. K. Mofrad, Daniel K. Nomura, Melanie Ott, Julia Schaletzky, Niren Murthy

**Affiliations:** a Graduate Program of Comparative Biochemistry, University of California Berkeley CA USA bajajtiya@berkeley.edu; b The Henry Wheeler Center of Emerging and Neglected Diseases 344 Li Ka Shing Berkeley CA USA jschaletzky@berkeley.edu; c Gladstone Institute of Virology Gladstone Institutes San Francisco CA USA melanie.ott@gladstone.ucsf.edu; d Chemical Biology Graduate Program, University of California Berkeley CA USA; e Department of Bioengineering, University of California Berkeley CA USA nmurthy@berkeley.edu; f Department of Mechanical Engineering, University of California Berkeley CA USA; g Department of Chemistry, University of California Berkeley CA USA; h Department of Medicine, University of California San Francisco CA USA; i Chan Zuckerberg Biohub San Francisco CA USA

## Abstract

The papain-like protease (PLpro) plays a critical role in SARS-CoV-2 (SCoV-2) pathogenesis and is essential for viral replication and for allowing the virus to evade the host immune response. Inhibitors of PLpro have great therapeutic potential, however, developing them has been challenging due to PLpro's restricted substrate binding pocket. In this report, we screened a 115 000-compound library for PLpro inhibitors and identified a new pharmacophore, based on a mercapto-pyrimidine fragment that is a reversible covalent inhibitor (RCI) of PLpro and inhibits viral replication in cells. Compound 5 had an IC_50_ of 5.1 μM for PLpro inhibition and hit optimization yielded a derivative with increased potency (IC_50_ 0.85 μM, 6-fold higher). Activity based profiling of compound 5 demonstrated that it reacts with PLpro cysteines. We show here that compound 5 represents a new class of RCIs, which undergo an addition elimination reaction with cysteines in their target proteins. We further show that their reversibility is catalyzed by exogenous thiols and is dependent on the size of the incoming thiol. In contrast, traditional RCIs are all based upon the Michael addition reaction mechanism and their reversibility is base-catalyzed. We identify a new class of RCIs that introduces a more reactive warhead with a pronounced selectivity profile based on thiol ligand size. This could allow the expansion of RCI modality use towards a larger group of proteins important for human disease.

## Abstract 2

Screening of the ChemDiv library against PLpro, an essential protein from the SCoV-2 virus, identified compound 5. Compound 5 is a reversible covalent inhibitor of PLpro and prevents SCoV-2 infection in cells.

## Graphical abstract

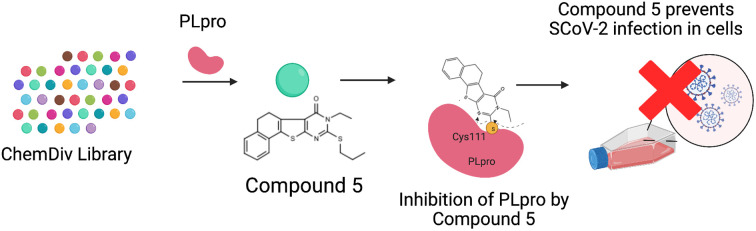

## Introduction

The severe acute respiratory syndrome coronavirus 2 (SCoV-2) has caused catastrophic levels of death and effective treatments are urgently needed.^1–4^ Small molecule therapeutics that can inhibit the RNA dependent polymerase (RdRp) and Main protease (Mpro) are clinically approved and had a big impact on reducing COVID-19 mortality.^5–8^ The success of these small molecule drugs has created a tremendous interest in developing inhibitors against other proteins from SCoV-2 that are also essential for viral replication.^9,10^

The papain-like protease (PLpro) from SCoV-2 is an essential protein for viral replication and an attractive target for developing small-molecule drugs.^11–14^ PLpro plays a crucial role in viral replication^15–17^ and prevents infected cells from generating interferons, which are essential for mounting an immune response against SCoV-2.^12,18,19^ PLpro cleaves the peptide sequence LxGG (x represents any amino acid), which is present in 3 sites in the immature SCoV-2 viral polyprotein. PLpro catalyzes the release of three non-structural proteins, termed nsp1, nsp2, and nsp3 from the immature viral polyprotein.^12^ Nsp1, nsp2, and nsp3 play critical roles in viral replication, and inhibition of PLpro blocks SCoV-2 replication in cells.^20^ PLpro also cleaves host proteins that contain the sequence RLRGG, which is present in several ubiquitin (Ub) and ubiquitin-like proteins (UbL), such as interferon-induced gene 15 (ISG15) proteins.^21^ PLpro has significant deISGylating and deubiquitinating activities and inhibition of PLpro induces the production of interferons by virally infected cells, which should lead to an enhanced immune response against the virus. Consequently, there is great interest in developing inhibitors against PLpro from SCoV-2.^14,20^

PLpro is a cysteine protease with a catalytic triad composed of histidine, cysteine, and aspartic acid, with 83% sequence homology to PLpro from SCoV and structural similarities to the deubiquitinating enzymes.^12^ Several crystal structures of PLpro have been solved,^22^ and these studies have revealed that it binds Gly–Gly in the first two positions of its peptide binding site, and does not have a well-defined binding pocket near its active site, in contrast to other proteases that need to accommodate peptides with larger side chains.^23^ PLpro is a challenging protein to drug due to its ill-defined binding pocket, and progress towards developing PLpro inhibitors has been slow despite its great antiviral potential.^3,20,24,25^

Several high throughput screens (HTS) have been performed against PLpro from SCoV and SCoV-2, and these studies have generated pharmacophores that can inhibit PLpro and viral replication in cells.^26,27^ The compound GRL0617 and its derivatives are the best characterized class of PLpro inhibitors. GRL0617 was identified in a 50 080-molecule screen on PLpro from SCoV.^28^ GRL0617 inhibited SCoV PLpro with an IC_50_ in the low micromolar range and can inhibit viral replication in cells. GRL0617 also inhibits PLpro from SCoV-2 and viral replication in cells, with IC_50_s in the micromolar range, and shows moderate antiviral activity against SCoV-2 in mice after oral delivery.^11,22,25–27,29^ GRL0617 has been further optimized against PLpro from SCoV-2, *via* structure-based drug design strategies, and its derivatives inhibit PLpro with nanomolar efficacy *in vitro* and inhibit viral replication in cells efficiently.^22–24,26,27,30,31^

Additional HTS screens on PLpro from SCoV-2 have generated other non-GRL0617 based pharmacophores that are promising leads.^26,32^ For example, Yuan *et al.* screened a 50 080 large compound library and identified a new class of PLpro inhibitors, based upon the fragment 5-oxo-1-thioxo-4,5-dihydro[1,3] thiazolo[3,4-*a*]quinazoline-3-carboxamide, which was able to inhibit PLpro from multiple corona viruses and inhibited SCoV-2 viral replication in hamsters and MERS-CoV in mice, and outperformed GRL0617 in animal studies.^33^ These experiments demonstrate the great potential of non-GRL0617 based chemical scaffolds. There are currently very few non-GRL0617 based scaffolds that can inhibit PLpro and viral replication in cells^20,32^ and alternatives to GRL0617 and its derivatives are greatly needed, given the high failure rate of small molecule therapeutics in clinical trials.

In this report, we screened a 115 000-molecule chemical library against PLpro from SCoV-2 and discovered a unique mercaptopyrimidine based pharmacophore, compound 5, which inhibits PLpro *in vitro* and inhibits SCoV-2 viral replication in cells. In addition to compound 5, we also identified several other compounds that were able to inhibit PLpro, which could serve as leads for further optimization. Finally, the mechanism by which compound 5 inhibits PLpro was also investigated by activity-based profiling, molecular dynamics, and a variety of other biochemical assays.

## Results and discussion

### Discovery of compounds inhibiting PLpro *via* high throughput screening

**Fig. 1:**
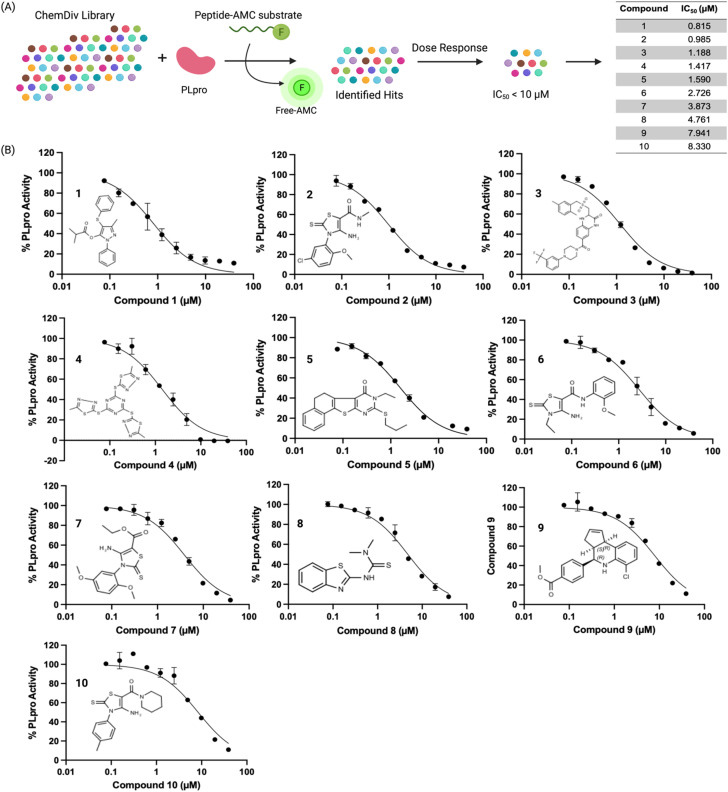
High throughput screening of a 115 000-compound library against PLpro identifies inhibitors with sub-micromolar IC_50_s. (A) PLpro was screened against the ChemDiv library and the IC_50_s of the 10 most effective hits are shown, their IC_50_s varied from 0.8 μM to 8.5 μM. (B) Dose response curves and chemical structures of the hits are shown. Four of the top hits (compound 2, 6, 7, and 10) shared the same electrophilic warhead based upon a cyclic thiocarbamate.

We used a fluorescent-based high throughput screening assay to identify inhibitors for PLpro from SCoV-2.^28^ This fluorescent assay uses RLRGG-AMC as a substrate for determining PLpro proteolytic activity. The release of the AMC was quantified by measuring the fluorescent intensity after exciting at 360 nm and measuring the emission at 460 nm. The PLpro domain of Nsp3 was recombinantly expressed and purified using a talon column. We optimized the protein and substrate concentration to 50 nM and 50 μM respectively, and our optimized assay had a *z*′ factor of 0.57 ± 0.05, suitable for HTS. We screened two libraries, termed the diverse and antibacterial libraries (115 000 compounds) at the concentration of 40 μM, and compounds that generated >50% inhibition were rescreened in duplicate, followed by a dose–response assay to determine the concentration that caused 50% PLpro inhibition (IC_50_) (Fig. 1A).

The preliminary screening of the diverse and antibacterial libraries resulted in 560 initial hits at a 3-sigma cutoff, and our screen had a hit rate of 0.48%. We further performed orthogonal assays to ensure that positive hits were not interacting directly with the fluorescence of the released coumarin-amine, reducing the hits to 211. A dose–response experiment was performed to determine the half maximal inhibitory concentration of the top 84 hits which revealed that ten compounds out of 115 000 were capable of inhibiting PLpro from SCoV-2 *in vitro* with IC_50_ < 10 μM (Fig. 1B). Several of the remaining hits were electrophiles and some were unique from inhibitors reported in previous articles. Four out of the ten compounds, compounds 2, 6, 7, and 10 share the same parent heterocycle structure and electrophilic warhead as the compound recently reported by Yuan *et al.*,^33^ which showed antiviral activity in cells and in animals, and this pharmacophore has great potential for further exploration. In addition, all of the compounds (1–10) are alkaloids, which have shown great potential as SCoV-2 antiviral agents.^34^

### Compound 5 exhibits anti-SCoV-2 activity

**Fig. 2:**
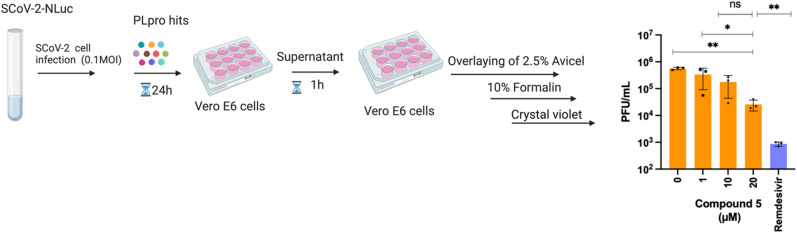
Compound 5 inhibits SCoV-2 replication in cells. Schematic diagram of viral plaque forming assay and data obtained with compound 5 in this assay. Vero E6 cells were infected with SCoV-2 virus, treated with compound 5 at various concentrations (1, 10, 20 μM) and a viral plaque forming assay was performed. Compound 5 reduced infectious virus levels by 3 and 22-fold at 10 μM and 20 μM concentrations, respectively.

To test antiviral activity, the top hits were screened on nano luciferase (NLuc) SCoV-2 infected TMRPSS2-expressing Vero E6 cells at various concentrations (1, 10, or 20 μM). Of the compounds tested, one compound exhibited antiviral activity in a dose dependent manner. Compound 5 reduced the NLuc levels of infected cells by 3-fold at 10 μM and 7-fold at 20 μM (data not shown). To validate the antiviral activity of compound 5, a separate antiviral study was performed with compound 5 using a plaque forming assay. The viral plaque forming assay validated the antiviral activity of compound 5 and compound 5 caused a 3 and 22-fold reduction in infectious virus levels at 10 μM and 20 μM concentrations, respectively. Remdesivir (100 μM) served as a positive control in this assay and potently inhibited the virus (Fig. 2). Finally, to ensure that the antiviral activity of compound 5 was not due to cytotoxicity, we performed a cell viability assay using the resazurin assay. Compound 5 was tested at four concentrations (1, 10, 25, 50 μM) in Vero CCL-81 cells and the cell viability at 1, 10 and 25 μM of compound 5 was greater than 85%. However, compound 5 had significant toxicity at a 50 μM concentration (Fig. S1†).

### Compound 5 is a covalent PLpro inhibitor

**Fig. 3:**
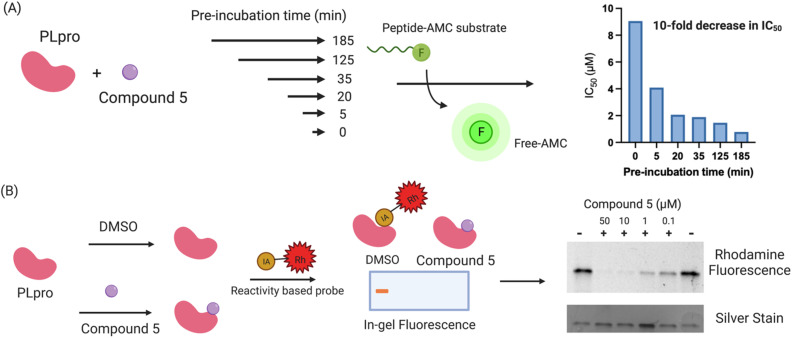
Compound 5 is a covalent inhibitor of PLpro. (A) The IC_50_ of compound 5 decreases by 10-fold after increasing the pre-incubation to 3 hours. Compound 5 was pre-incubated with PLpro for various times and the IC_50_ was measured. The IC_50_ of compound 5 decreased from 9 μM (no pre-incubation) to 0.9 μM after a 3 hour pre-incubation, suggesting that it is a covalent PLpro inhibitor; (B) activity based profiling of compound 5 demonstrates that it reacts with the cysteines of PLpro. PLpro was mixed with compound 5 and then chased with iodo-acetamide and compared with PLpro mixed with iodo-acetamide. Compound 5 prevents PLpro from reacting with iodo-acetamide and inhibits the fluorescent staining of PLpro, demonstrating that it reacts with thiols on PLpro.

Compound 5 contains a mercaptopyrimidine fragment, which can potentially undergo addition–elimination reactions with nucleophiles. For example, Murugesan *et al.* recently identified a mercaptopyrimidine based inhibitor of tuberculosis cell growth, which reacted with glutathione under physiological conditions.^27,35^ PLpro is a cysteine protease with a nucleophilic cysteine in its active site, which could potentially react with compound 5*via* an additional elimination reaction. We performed a time dependent PLpro inhibition assay with compound 5 to determine if pre-incubation time lowered its IC_50_. Pre-incubation time lowers the IC_50_ of covalent inhibitors because it gives more time for the protein to react with the inhibitor. In contrast, pre-incubation time frequently has no effect on the IC_50_ of non-covalent inhibitors because their *k*_off_s are generally on the timescale of second to minutes. PLpro was preincubated with compound 5 for various times ranging from 5 minutes to 3 hours and the IC_50_ of compound 5 was measured for each pre-incubation time point. The IC_50_ of compound 5 decreased by 10-fold after pre-incubation with PLpro for 3 hours (Fig. 3A), suggesting it is a covalent inhibitor.

We performed activity-based profiling experiments to determine if compound 5 covalently reacted with the cysteines or lysines of PLpro.^36^ Iodoacetamide-rhodamine was used as the probe for cysteine reactivity analysis and NHS-rhodamine was the probe for investigating lysine reactivity on PLpro. A traditional pulse chase experiment was performed with PLpro and the rhodamine dyes, where PLpro was first incubated with compound 5 and then incubated with iodoacetamide-rhodamine or NHS-rhodamine. After the dye incubation, PLpro was run on a protein gel and imaged *via* fluorescence. Compound 5 was able to prevent PLpro from reacting with iodo-acetamide, demonstrating that it reacts with nucleophilic thiols on PLpro, most likely the catalytic cysteine in the active site (Fig. 3B). The other thiols in PLpro are either disulfides, which are unreactive to nucleophiles, or zinc chelated and have diminished nucleophilicity. In contrast to iodoacetamide, compound 5 was unable to prevent PLpro from reacting with NHS-rhodamine, demonstrating that it is not reacting with lysine residues.

### Compound 5 is a reversible covalent inhibitor of PLpro

Compound 5 contains a mercaptopyrimidine fragment, which can potentially react with PLpro in a reversible manner *via* the addition–elimination reaction pathway. Reversible covalent inhibitors (RCIs) have great potential as electrophilic warheads because they combine the high efficacy of covalent inhibitors with the low toxicity of non-covalent inhibitors.^37^ In addition, RCIs are less immunogenic than covalent inhibitors and their on-target residence time and therapeutic effectiveness can be fine-tuned by modulating their binding affinity with the target protein.^38^ However, developing RCIs is challenging because there are very few electrophiles that reversibly react with biological nucleophiles under physiologic conditions. At present, Michael addition acceptors are the only class of electrophilic warheads that can generate RCIs, and this limits the development of RCIs because numerous proteins do not react with the relatively mild/inert Michael acceptors. Compound 5 does not contain a Michael acceptor but has the potential to be an RCI because of the relatively low p*K*_a_ of the thiol fragment attached to the pyrimidine ring, which could potentially be displaced by an incoming nucleophile.

**Fig. 4:**
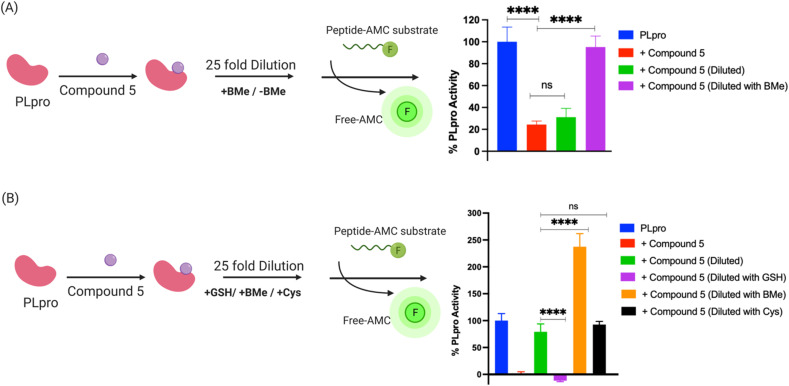
Compound 5 is a reversible covalent inhibitor of PLpro and its reversibility is triggered by exogenous thiols. (A) Schematic diagram of jump dilution assay and data obtained with compound 5. Compound 5 was mixed with PLpro and diluted in the presence of beta-mercaptoethanol (BMe) or PBS and assayed for PLpro inhibition. Compound 5 still inhibits PLpro after dilution with PBS. In contrast, dilution in the presence of BMe causes 90% recovery of PLpro activity, demonstrating that compound 5 is a reversible PLpro inhibitor and its reversibility is triggered by exogenous thiols; (B) the reversibility of the compound 5-PLpro adduct is determined by the size of the exogenous thiol. A schematic diagram of the jump dilution assay. BMe and cysteine can regenerate active PLpro from the PLpro-compound 5 adduct, whereas reduced glutathione (GSH) cannot.

To investigate if compound 5 constitutes a novel class of RCI, we performed a jump dilution assay with PLpro, compound 5 and beta-mercaptoethanol (BMe). PLpro and compound 5 were mixed and allowed to react, diluted 25-fold in the presence of 5 mM BMe^38,39^ and the inhibition of PLpro was measured. We observed that PLpro recovered 90% of its activity after 5 minutes dilution with 5 mM BMe (Fig. 4A), in contrast dilution in phosphate buffer saline (PBS) had no effect on the activity of PLpro.

BMe is not a biological nucleophile, and we re-did the jump dilution assay in the presence of either 5 mM reduced glutathione (GSH) or 200 μM cysteine (their physiological concentrations)^40^ to determine if the PLpro-compound 5 adduct would rapidly decompose in cells. We observed that the addition of cysteine recovered the activity of PLpro, however GSH did not (Fig. 4B). These experiments suggest that compound 5 is an RCI and its reversibility is based upon the size of the displacing thiol. Glutathione is larger than cysteine and BMe and presumably cannot access the active site of PLpro and therefore does not displace compound 5 from PLpro. In contrast, smaller thiols such as BMe and cysteine can apparently access the PLpro active site and can displace compound 5. Collectively, these experiments demonstrate compound 5 is an RCI and that the mercaptopryimidine ring can act as a new scaffold for generating RCIs that are not based upon Michael addition reactions. In addition, the mercaptopryimidine ring can function as a size selective filter for thiols and this unique feature should allow it to find numerous applications in drug discovery.

### Compound 5 tolerates modifications of its mercapto group

**Fig. 5:**
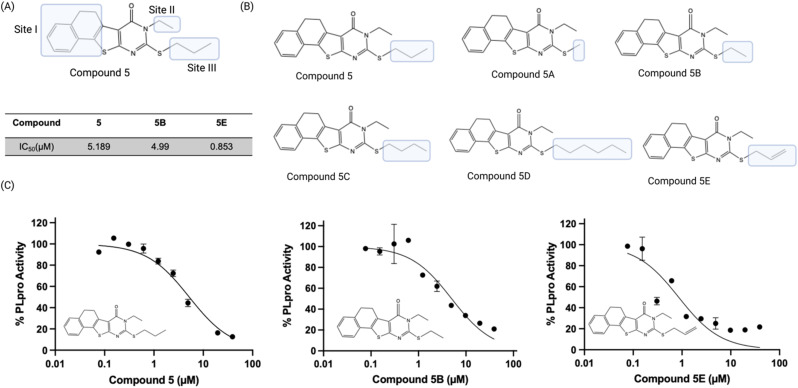
SAR of compound 5 demonstrates it that tolerates modification at its mercapto group. (A) Compound 5 derivatives containing modifications at site I, site II, and site III were evaluated; (B) only modification at the mercapto site in the pyrimidine ring is tolerated. 5E had an IC_50_ of 0.9 μM and had its mercapto site modified with an allyl fragment; (C) dose response curves of compounds 5, 5B and 5E.

**Fig. 6:**
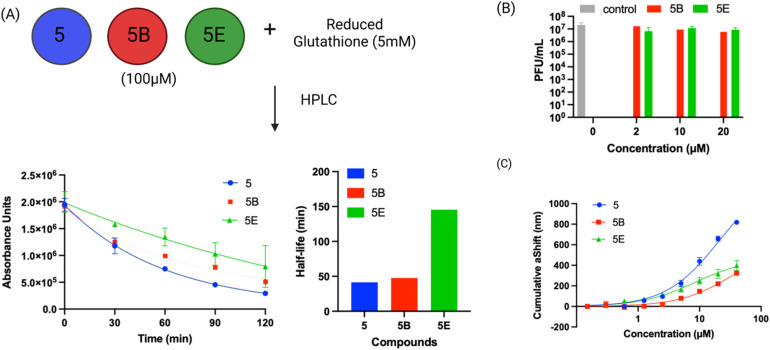
Compound 5 has low reactivity with thiols and binds PLpro with micromolar affinity. (A) The reaction half-life of compound 5 and its active derivatives was determined in the presence of GSH, their half-lives varied from 41–145 minutes; (B) plaque assay for compound 5 analogs (5B and 5E) show that analogs do not exhibit antiviral activity; (C) the binding constant of PLpro with compound 5 was determined *via* surface plasmon resonance. The binding constants (*K*_D_) of compound 5 derivatives correlates with their IC_50_s and varied from 36 to 8 μM.

We performed a preliminary hit exploration of compound 5 derivatives to identify sites in compound 5 that could be modified to enhance its activity. Compound 5 derivatives with modifications at site I, site II and site III were investigated for their ability to inhibit PLpro activity (Fig. 5A). Compound 5 does not tolerate modifications at site I and site II, and these derivatives were largely inactive. In contrast, modifications at site III were tolerated and resulted in several derivatives that were more active than compound 5 (Fig. 5B). For example, compound 5E contained an allylic thiol instead of a propyl thiol and had an IC_50_ of 0.9 μM, which was approximately 5 times lower than compound 5's. In addition, compound 5B, which had an ethyl substituent on the thiol was also tolerated and had an IC_50_ of 5.0 μM and was almost identical to compound 5 (Fig. 5C). Substituents larger than a propyl group on the thiol, such as butyl, were not tolerated and resulted in almost a complete loss of activity. Although compounds 5B and 5E acted as PLpro inhibitors, they did not have any antiviral activity in Vero E6 cells as determined by a plaque assay (Fig. 6B).

### Compound 5 has low non-specific reactivity with thiols

Compound 5 and the mercaptopyrimidine fragment represent a new under-explored class of electrophiles with numerous potential applications given their biological activity. The thiol reactivity of mercaptopyrimidines under physiologic conditions has never been investigated. We determined the half-life of compound 5 and its derivatives in the presence of 5 mM GSH *via* HPLC to gauge its reactivity in comparison to other electrophiles commonly used in generating covalent inhibitors. The GSH half-lifes of compounds 5, 5B and 5E varied between 41.0 minutes to 145.4 minutes (Fig. 6A) and are in a similar range to phenyl acrylamide-based electrophiles (*t*_½_ of 179 minutes with 5 mM GSH) which are a commonly used scaffold for generating covalent inhibitors.^41^ For example, the clinically approved covalent kinase inhibitors afatinib, neratinib and osimertinib are all based upon phenyl acrylamide-based electrophiles.

### Surface plasmon resonance (SPR) analysis of compound 5 and its derivatives

We performed SPR analysis of compound 5 and its derivatives with PLpro to determine their binding affinity with PLpro. His-tagged PLpro was immobilized on Ni-NTA sensor chips and various concentrations of compound 5 and its analogs (5B and 5E) were applied to the chip and the plasmon responses were recorded. The SPR results obtained with compound 5 and its analogs demonstrate that these compounds can bind PLpro and their binding affinity correlates with their IC_50_s. For example, 5E had an inhibition IC_50_ of 0.85 μM and had a *K*_D_ of 8.3 μM, and compound 5 had an IC_50_ of 5.0 μM and had a *K*_D_ of 21 μM. The allyl modification of 5E is the only difference between compounds 5 and 5E and appears to significantly enhance 5E's interaction with PLpro.

### Molecular dynamics simulation of compound 5 with PLpro

**Fig. 7:**
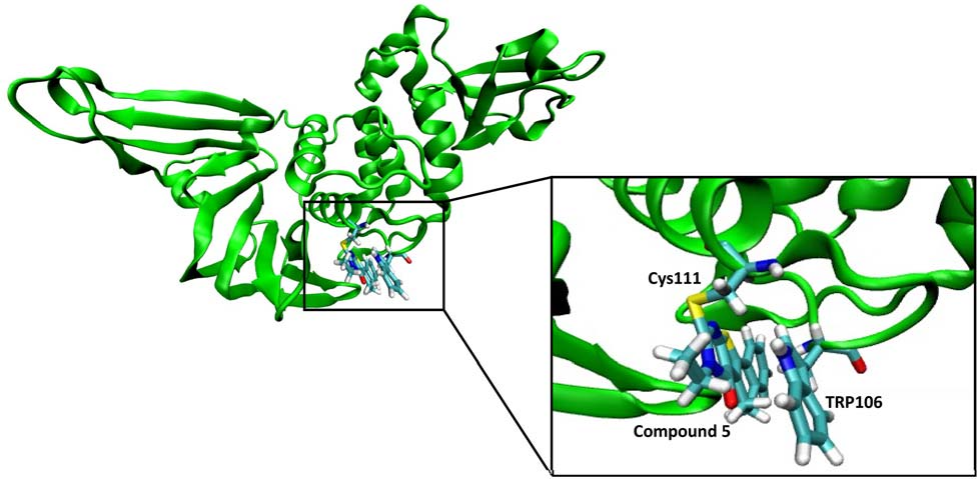
Molecular dynamic studies of compound 5 with PLpro. Compound 5 reacts with Cys111 in the active site of the PLpro and interacts with Trp106 *via* pi–pi stacking.

We performed a molecular dynamics simulation of compound 5 with the active site of PLpro to obtain insight into the potential interactions it may have with the active site. Compound 5 was covalently tethered to the active site cysteine of PLpro and an MD simulation was run, three independent times for 90 ns each. After a few picoseconds of simulation all three simulations generated structures that had compound 5 turned towards Trp106 and it interacted with Trp106 *via* π–π stacking for most of each simulation (Fig. 7).

## Experimental

### Materials and methods

#### Expression and purification of PLpro

The papain-like protease (PLpro) expressing plasmid, 2BT-nsp3-PLpro was a kind gift from Ott lab at Gladstone institute, UCSF. The plasmid was transformed into the *E. coli* BL21 (DE3) and plated on an ampicillin resistant LB agar plate. Next day, a colony was picked up for overnight culture in presence of ampicillin 100 μg mL^−1^. For large-scale protein purification, a 1 L culture of 2XYT media was grown using overnight culture (1 : 100) at 37 °C (210 rpm). The bacterial culture was grown to OD_600_ ∼ 0.8–1.0 and induced with 1 mM IPTG. The protein was expressed at 20 °C for overnight (18–20 hours). Bacterial culture was harvested at 4000*g*, and cell pellets were resuspended in 30 mL lysis buffer (25 mM Tris–HCl pH 8.0, 250 mM NaCl, 10% glycerol, 5 mM BMe), supplemented with protease inhibitor tablets (Pierce). The cell culture was sonicated at 20% amplitude for 7 minutes (0.5 s ON, 1.5 s OFF). Cellular debris was pelleted down by centrifuged at 15 000*g* for 20 minutes at 4 °C. Supernatant was loaded on Talon column (GE Healthcare Life Sciences) (pre-equilibrated with lysis buffer) at speed of 1 mL min^−1^. Non-specific proteins were washed with 20 column volumes of buffer A (lysis buffer supplemented with 25 mM imidazole). PLpro protein was eluted with 5 column volumes of buffer-B (lysis buffer supplemented with 250 mM imidazole). The eluted protein was concentrated using 10 kDa MWCO filter (Amicon-Millipore) up to 2 mg mL^−1^.

#### Fluorescent assay to determine the activity of the proteins

The kinetic assays were developed in 384 well plate to optimize the assay conditions (protein and substrate concentration, and incubation times) as described in Ratia *et al.*^28^ In brief description, the assays were set up with different concentration of protein (0–100 nM), different concentration of substrate (0–200 μM) and the fluorescent emission intensity was measured at different time intervals (0–30 minutes). The final reaction volume of 50 μL consisted of 30 μL of buffer (20 mM Hepes pH 7.5, 100 mM NaCl, 0.1% mg mL^−1^ BSA), 10 μL of protein and 10 μL substrate. The fluorogenic peptide substrate Arg-Leu-Arg-Gly-Gly-AMC (Z-RLRGG-AMC) (Bachem Biosciences) acted as reaction initiator and fluorescent emission intensity was measured at given wavelengths (excitation: 360 nm and emission: 460 nm) at time intervals.

#### High throughput screening

We developed a high throughput assay to screen a 115 000-compound library at UC Berkeley Drug Discovery Center (DDC) at the Center of Emerging and Neglected Diseases. The assay was optimized for 384 well black plates (corning 3573) with the total reaction volume of 25 μL, with equal volumes of protein and substrate (12.5 μL) and 0.5 μL of DMSO or compound (final concentration of 40 μM) dissolved in DMSO which was pre-plated with an Analytik-Jena Cybio liquid handler which was also used to add protein and substrate reagents later during the actual run. Protein and substrate were diluted in same buffer used in 4.2 with exception of Antifoam (Spectrum chemicals, Cat# A1302) with the ratio of 1 : 5000 that was added to reduce surface tension. The fluorescent emission intensity was measured at the intervals of 0, 10, 20, 30 and 60 minutes using a 2104 Envision reader (PerkinElmer; excitation: 360 nm and emission: 460 nm). The 60 minute time point yielded the best Z prime and was chosen as the end point for all screening. The data was analyzed using dose response curve models (4 parameter fit).

#### Hit confirmation and dose response

Hits from primary screen were cherry-picked on a Tecan Freedom EVO 150 for rescreening to confirm. Confirmation was done at 40 μM in duplicate and a 10 point 1 : 2 serial dilution for dose response starting at 40 μM was also run in duplicate. Along with the dose response assays, we performed an orthogonal assay to rule out false positives. The orthogonal assay included the substrate and compounds with buffer only (without protein).

#### Structure–activity relationship (SAR)

The compounds and analogs were purchased from ChemDiv. Both activity assays, fluorescent and dose response, were repeated with these compounds in two additional conditions, 0.01% Triton-X and 1 mM reduced glutathione (mimicking the cellular reducing conditions).

#### Cytotoxicity assay

Vero CCL-81 cells (provided by UC Berkeley Cell culture facility) were thawed at 37 °C and were grown in Dulbecco's Modified Eagle Medium (DMEM, high glucose and pyruvate) (Gibco) in 100 mm dish. The media was supplemented with 10% fetal bovine serum (FBS) and 1% antibiotic and antimycotic (100 U mL^−1^ penicillin and 100 μg mL^−1^ streptomycin). The cells were split into 96 well plate with the count of 10 000 cells per well. Each compound was added in well with different concentration (1, 10 and 20 μM) with the 0.5% of DMSO and incubated for 72 hours. After 72 hours, resazurin (10 μg mL^−1^) was added and fluorescence intensity was measured at excitation: 560 nm and emission: 590 nm. The experiment was performed in triplicates.

#### Mammalian cell lines and culture conditions

Vero-E6 were procured from ATCC were cultured in DMEM (Corning) supplemented with 10% fetal bovine serum (FBS) (GeminiBio), 1% glutamine (Corning), and 1% penicillin–streptomycin (Corning) at 37 °C, 5% CO_2_.

#### SCoV-2 virus culture

SCoV-2 isolate SCoV-2-NLuc and USA-WA1/2020 (BEI NR-52281) were used for all infection studies. The virus infection experiments were performed in a Biosafety Level 3 laboratory. Working stocks of SARS-CoV-2 were made in TMPRESS-2 expressing Vero-E6 cells and were stored at −80 °C until used.

### Viral infection studies

#### SCoV-2 NLuc antiviral assay

Vero cells were seeded (12 000 cells per well) in a white opaque 96-well plate. After overnight incubation the cells were infected with SARS-CoV-2-NLuc at 0.01 multiplicity of infection (MOI). At 1 hour post infection (hpi) the working stock of the virus was replaced by multiple concentrations (20, 10 and 0.1 μM) of compounds. Remdesivir and DMSO were used as positive and negative controls respectively. At 24 hpi, 50 μL of Nano luciferase substrate (Promega) was added to each well and after 10 min of incubation at room temperature luciferase signals were measured using a Promega Glow Max microplate reader. The relative luciferase signal was recorded and plotted against compound concentration using software Prism.

#### SCoV-2 USA-WA1/2020 antiviral assay

Vero cells were seeded in a 12 well plate. After overnight incubation the cells were infected by SARS-CoV-2 USA-WA1/2020 strain at MOI of 0.1. The media was replaced by multiple concentrations (20, 10 and 0.1 μM) of compound B. Remdesivir and DMSO were used as positive and negative controls respectively. The cells were further incubated at 37 °C and 5% CO_2_ for 24 hours and culture supernatant were harvested for plaque assay.

#### Plaque-forming assays

Culture supernatants at 24 hpi were used for plaque assay. Vero cells were seeded (0.2 × 10^6^ cells per well) in 12 well plates and after overnight incubation the cells were infected with differential concentrations of supernatants from test and control groups. After 1 h absorption period, the media in the wells was overlaid by 2.5% Avicel (Dupont, RC-591) and incubated for 72 hours. After incubation the Avicel was removed and cells were fixed in 10% formalin for one hour and stained with crystal violet for 10 minutes, for visualization of plaque forming units per ML.

#### Mechanism of inhibition

The PLpro protein (50 nM) was preincubated with different concentration of inhibitor (0, 1, 2.5, 5, 10 and 20 μM) for different time points (0, 5, 10, 20, 35, 95, 125, 185 minutes). The substrate (50 μM) was added and measured the fluorescence. The data was analyzed using Enzyme – inhibition, GraphPad Prism.

#### Half-life determination of compound 5 and its analogs

The half-life of compound 5 was determined using HPLC. 100 μM of compound 5 and its analogs was incubated with 5 mM reduced glutathione at pH 7.4 for different time intervals (0, 30, 60, 90, 120 and 180 minutes). The positive control sample was run without reduced glutathione. The half-life was calculated using first-order reaction kinetic equation, *t*_1/2_ = 0.693/*k*.

#### Activity based protein profiling

For gel-based ABPP experiments with PLpro, pure SCoV2 PLpro protein (0.1 μg per sample for IA-rhodamine and 0.05 μg for NHS-rhodamine) was pretreated with either DMSO vehicle or desired dosage of compound 5 at 37 °C for 1 hour in 25 μL PBS. Samples were subsequently treated with either 100 nM tetramethylrhodamine-5-iodoacetamide dihydroiodide (IA-rhodamine) (Setareh Biotech 6222) or 500 nM 5/6-carboxy-tetramethyl-rhoadmine succinimidyl ester (NHS-Rhodamine) (Thermo Scientific™ 46406) protected from light at room temperature for 1 hour. Samples were incubated with 10 μL 4× Laemmli sample buffer, boiled at 95 °C for 10 min, and separated by SDS/PAGE. Probe-labeled proteins were analyzed by in-gel rhodamine fluorescence using a ChemiDoc MP (Bio-Rad). Protein loading was assessed by Silver Staining.

#### Jump dilution assay

PLpro was mixed with compound 5 at the 10-fold higher concentration of IC_50_ (10 × IC_50_) and allowed to form protein-inhibitor complex at saturating conditions at room temperature. The complex was then rapidly diluted in a buffer supplemented with 5 mM reduced glutathione, 5 mM beta-mercaptoethanol (BMe), 200 μM cysteine to bring the compound's concentration of 1/10 × IC_50._ After 5 minutes of dilution, the substrate was added and incubated for 30 minutes. The fluorescent intensities were read at the excitation wavelength of 360 nm and emission wavelength of 460 nm.

#### Molecular dynamic studies

To study molecular interactions between PLpro active site and the inhibitor, first we prepared chemical structure of covalent inhibitor by drawing it in MarvinSketch (ChemAxon 2019) and has been modified according to its final state after covalent bonding. For Molecular Dynamics (MD) simulation using GROMACS,^42^ we used CHARMMS36 (ref. 43) forcefield. So, to get the forcefield parameters of the inhibitor, we convert 2D structure into 3D using OpenBable^44^ software and transferred them into CGENFF^43,45–47^ online server. Protein model of SARS-CoV-2 PLpro (PDB: 6W9C) was obtained from RCSB server. After preparing all forcefield parameters, we defined covalent inhibitor as a new residue by manually adding its parameters in the CHARMM36 residue definition file. Moreover, we needed to modify Cys111 of PLpro as well, to turn it into its final state after covalent bonding by changing its residue type from Cys to Cys2. Finally, we applied corresponding sulfur–carbon bond parameters by manually inserting them into the bonded forcefield parameters of CHARMM36.

After defining all parameters, the energy minimized inhibitor with 1-ClickDocking online server (https://mcule.com) is brought in vicinity of CYS111 using VMD.^48^ The resulting protein complex were placed inside a water box with 13 nm side, ensuring minimum 2.5 nm distance between protein complex and walls to minimize any cross talk among protein and its images. Note, water molecules were modeled using TIP3P forcefield. Next, we neutralized the simulation box with Na^+^ and Cl^−^. Long range electrostatic interactions were captured by Particle Mesh Ewald (PME) method.^49^ To model molecular interactions, we started with system energy minimization, then we did *NVT* and *NPT* simulations for 100 ps to equilibrate system with V-rescale thermostat (modified Berendsen thermostat) and Berendsen barostat.^50^ Finally, we did MD production simulations for 30 ns (time step of 2 fs) with V-rescale thermostat and Parrinello-Rahman barostat^51^ and repeated modeling two more times (90 ns in total) to capture statistical behavior. It should be noted that all resulting trajectories were visualized and analyzed with VMD tools.

## Conclusions

PLpro inhibitors have great potential for improving the treatment of SCoV-2. PLpro inhibitors inhibit viral infection *via* multiple methods and block viral replication and suppress the production of interferons by infected cells. Up-regulating the production of interferons by SARS-CoV2 infected could have synergistic effects with inhibiting viral replication because it will prevent neighboring cells from being infected with viruses. SCoV-2 has evolved to contain multiple proteins that reduce the production of interferons and enable immune cells evasion, and it is likely that these pathways play essential roles in allowing SCoV-2 to spread efficiently. However, despite their promise developing PLpro inhibitors has been challenging. HTS on PLpro have yielded very few promising leads and existing PLpro inhibitors have shown minimal activity in mice. In contrast, in the case of Mpro inhibitors, multiple classes of inhibitors have been developed that have been successful in a wide variety of animal models and in human clinical trials. There is consequently a great need for the development of new PLpro inhibitors.

In this report, we screened a 115 000-molecule library and identified a new chemical scaffold based on a mercaptopyrimidine fragment that inhibited PLpro activity with IC50s in the low micromolar range, was an RCI, and also inhibited viral replication in cells. Compound 5 is to our knowledge the first example of an RCI that can inhibit PLpro and viral replication in cells. Compound 5 undergoes a nucleophilic aromatic substitution (S_N_Ar) reaction with thiols in PLpro and reacts in a fundamentally different mechanism than traditional RCIs, which are based upon the reverse Michael addition reaction. RCIs have significant potential as therapeutics because of their ability to inhibit protein activity for long periods of time like covalent inhibitors, but do not induce the toxicity of covalent inhibitors because of their reversible nature. However, developing RCIs is challenging because of the limited number of electrophiles that form reversible bonds with proteins. The mercaptopyridine fragment represents a new scaffold for developing RCIs and should enable the development of RCIs that inhibit the function of new classes of proteins outside of PLPro, which do not perform Michael addition reactions efficiently. In this report we also performed an SAR of compound 5 and identified analogs with lower IC_50s_ and higher stability in the presence of GSH. Collectively, these experiments demonstrate that compound 5 is a promising lead fragment for future development given its efficacy in cells and ability to act as an RCI.

## Author contributions

Conceptualization, N. M., J. S., M. O., D. N. and M. R. K. M.; methodology, T. B., E. W., R. K. S, E. K., K. S. P., K. B., Z. K., U. S.-G., G. R. K.; software, T. B. and N. M.; validation, T. B., N. M.; formal analysis, T. B., E. W., R. K. S, E. K., K. S. P., K. B., Z. K., G. R. K.; investigation, T. B. and N. M.; resources, N. M., J. S., M. O., D. N., M. R. K. M.; data curation, N. M. and T. B.; writing – original draft preparation, T. B. and N. M.; writing – review and editing, N. M., T. B., J. S., G. R. K., R. S.; visualization, T. B.; supervision, N. M., J. S., M. O.; project administration, N. M.; funding acquisition, N. M., M. O., J. S. All authors have read and agreed to the published version of the manuscript.

## Conflicts of interest

There are no conflicts to declare.

## Supplementary Material

RA-013-D3RA01915B-s001
