# Supplementary material for: Mercapto-pyrimidines are reversible covalent inhibitors of the papain-like protease (PLpro) and inhibit SARS-CoV-2 (SCoV-2) replication
Source: RSC Adv. 2023 Jun 12;13(26):17667–77. doi: 10.1039/d3ra01915b (PMC10259201; doi:10.1039/d3ra01915b)
Supplement: RA-013-D3RA01915B-s001 [file RA-013-D3RA01915B-s001.pdf]

Electronic Supplementary Material (ESI) for RSC Advances.
This journal is © The Royal Society of Chemistry 2023

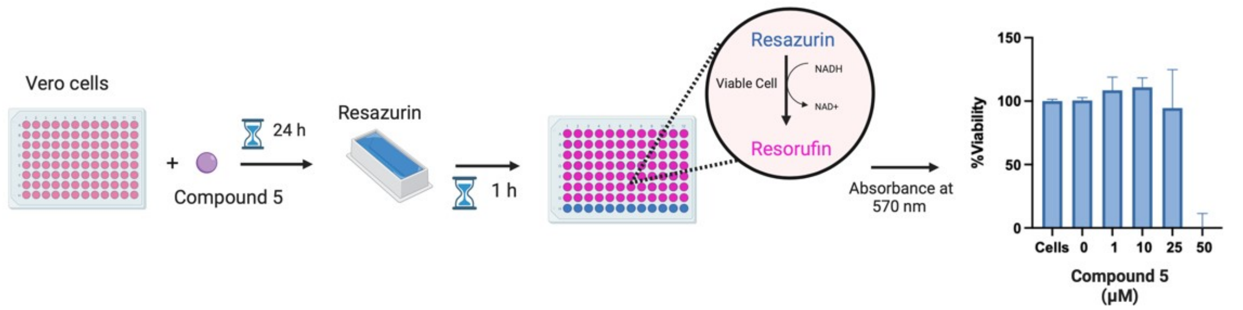

Fig S1. Cytotoxicity assay of compound 5. Different concentrations (1, 10, 20 and 50 μM) of the compound 5 were tested in Vero CCL81 cells. The viability was analyzed using resazurin assay and measured absorbance at 570 nm.

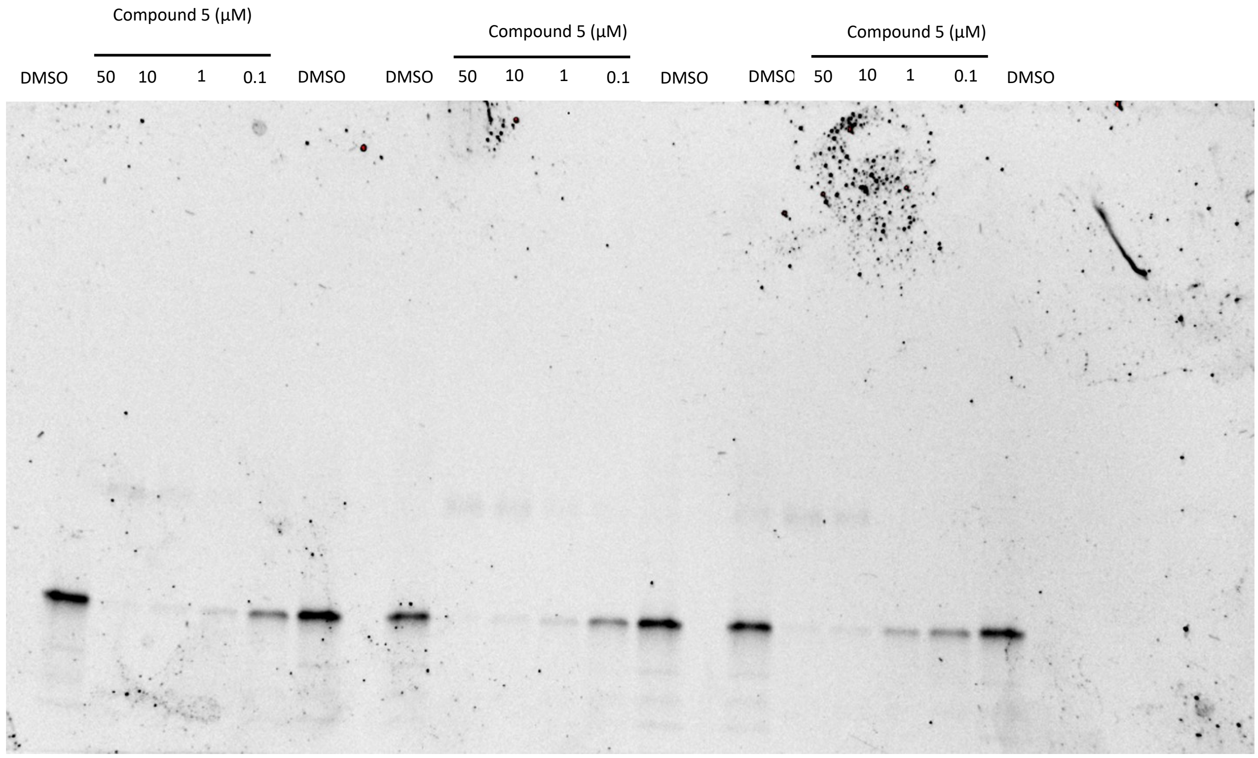

Raw image of Fig 3. Activity based protein profiling of compound 5. Cysteines in PLpro were chased with iodoacetamide-rhodamine in absence and presence of compound 5 (concentrations of 0.1, 1, 10 and 50µM). The gel was imaged using rhodamine fluorescence.

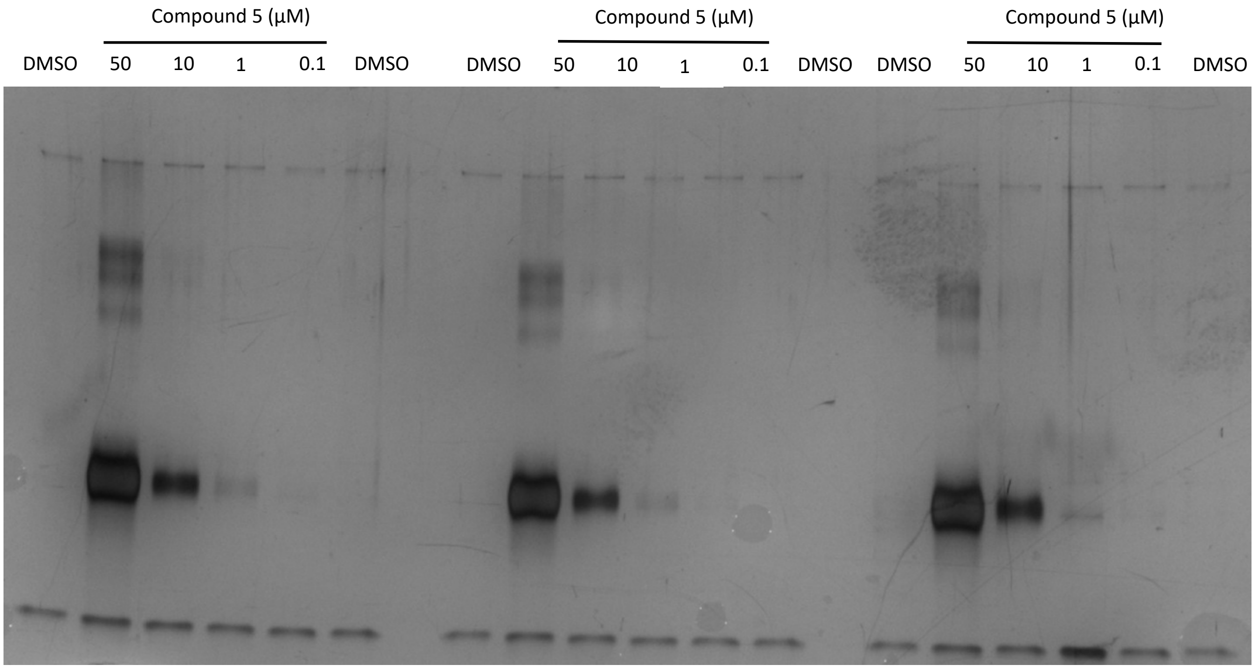

Raw image of Fig 3. Activity based protein profiling of compound 5. Cysteines in PLpro were chased with iodoacetamide-rhodamine in absence and presence of compound 5 (concentrations of 0.1, 1, 10 and 50µM). The gel was stained with silver stain for control.

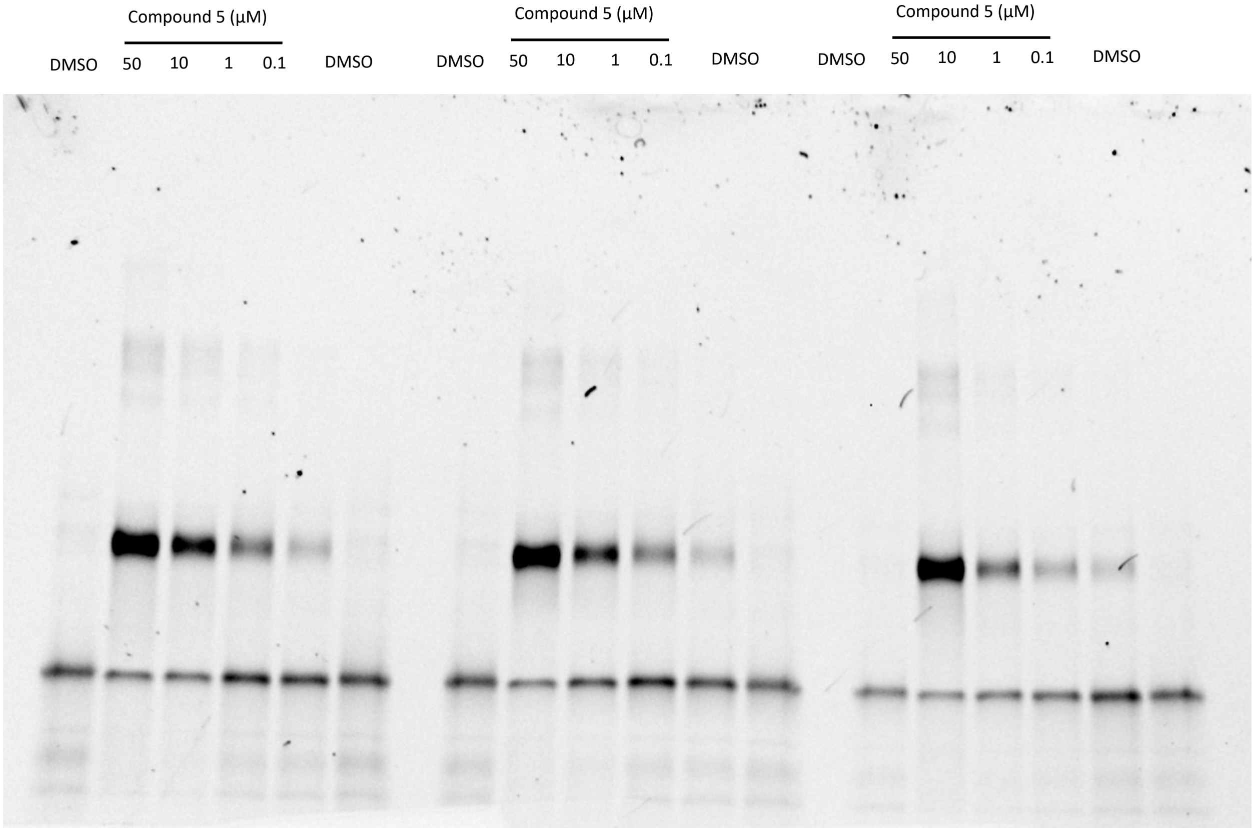

Raw image. Activity based protein profiling of compound 5. Lysines in PLpro were chased with NHS-rhodamine in absence and presence of compound 5 (concentrations of 0.1, 1, 10 and 50μM). The gel was imaged using rhodamine fluorescence.

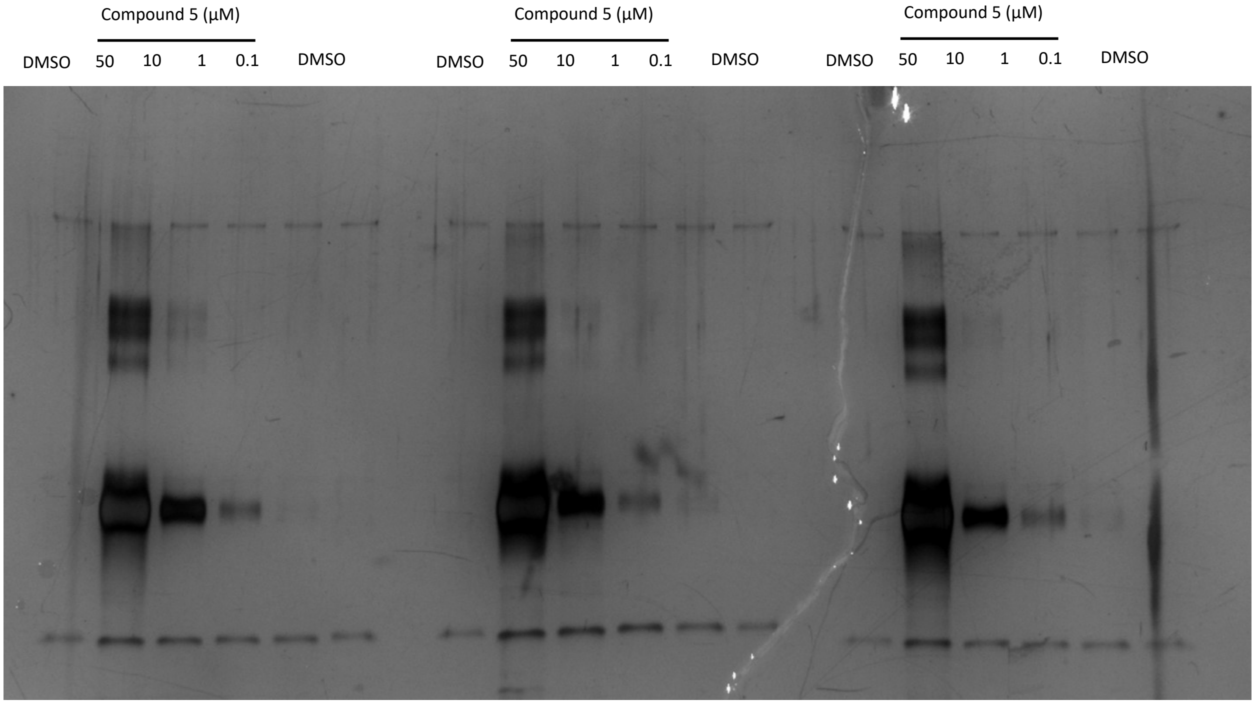

Raw image. Activity based protein profiling of compound 5. Lysines in PLpro were chased with NHS-rhodamine in absence and presence of compound 5 (concentrations of 0.1, 1, 10 and 50µM). The gel was stained with silver stain for control.
